# Feeding Ecology Informs Parasite Epidemiology: Prey Selection Modulates Encounter Rate with *Echinococcus multilocularis* in Urban Coyotes

**DOI:** 10.1371/journal.pone.0121646

**Published:** 2015-03-13

**Authors:** Stefano Liccioli, Carly Bialowas, Kathreen E. Ruckstuhl, Alessandro Massolo

**Affiliations:** 1 Department of Ecosystem and Public Health, Faculty of Veterinary Medicine, University of Calgary, 3280 Hospital Drive NW, Calgary, AB, T2N 4Z6, Canada; 2 Department of Biological Sciences, University of Calgary, 2500 University Drive NW, Calgary, AB, T2N 1N4, Canada; University of Minnesota, UNITED STATES

## Abstract

We investigated the role of urban coyote feeding ecology in the transmission of *Echinococcus multilocularis*, the causative agent of Alveolar Echinococcosis in humans. As coyotes can play a main role in the maintenance of this zoonotic parasite within North American urban settings, such study can ultimately aid disease risk management. Between June 2012 and June 2013, we collected 251 coyote feces and conducted trapping of small mammals (n = 971) in five parks in the city of Calgary, Alberta, Canada. We investigated *E*. *multilocularis* epidemiology by assessing seasonal variations of coyote diet and the selective consumption of different rodent intermediate host species. Furthermore, accounting for small mammal digestibility and coyote defecation rates we estimated the number of small mammal preys ingested by coyote and consequently, coyote encounter rates with the parasite. Dominant food items included small mammals, fruit and vegetation, although hare and deer were seasonally relevant. The lowest frequency of occurrence per scat of small mammals was recorded in winter (39.4 %), when consumption of deer was highest (36.4 %). However, highest encounter rates (number of infected hosts predated/season) with *E*. *multilocularis* (95% CI: 1.0 - 22.4), combined with the lack of predation on non-competent small mammal species, suggest that winter is the critical season for transmission and control of this parasite. Within the small mammal assemblage, voles (*Microtus pennsylvanicus* and *Myodes gapperi*) were the selected preys of urban coyotes and likely played a key role for the maintenance of the urban sylvatic life-cycle of *E*. *multilocularis* in Calgary.

## Introduction

The feeding ecology of wild species is traditionally investigated to assess predator-prey relations and dietary selectivity [1–3], habitat requirements [1–3], causes of decline [4] and human-related conflicts [5], overall aiming to identify conservation policies and inform management strategies. Much less frequently, animal feeding ecology is analyzed to shed light on the transmission of those parasites that depend on the predator-prey relationship between definitive and intermediate hosts [6].

Among trophically-transmitted parasites, *Echinococcus multilocularis* offers an interesting and complex system that may shed light on important ecological and epidemiological processes. This parasitic cestode is widely distributed in the Northern Hemisphere [7] and mainly infects wild canids such as foxes (*Vulpes* spp.) and coyotes (*Canis latrans*) as definitive hosts, and more than 40 species of small mammals (mainly Arvicolidae, Cricetidae and Muridae, [8]) as intermediate hosts [9]. Importantly, the parasite is the causative agent of Alveolar Echinococcosis in humans, currently considered among the most serious zoonotic diseases of the Northern hemisphere (case fatality rate >90% if untreated [8]). Typically, infections in humans follow accidental ingestion of parasite eggs through contaminated soil and/or food, or through contact with infected dogs [10]. Despite a primarily sylvatic life-cycle, *E*. *multilocularis* can circulate and be maintained within urban habitats [8, 10]: here, given the high risk of zoonotic transmission, understanding parasite ecology becomes crucial for disease prevention and risk management.

In the Northern-central region of North America (13 US states and the four Canadian western provinces [11–13]), meadow voles (*Microtus pennsylvanicus*) and deer mice (*Peromyscus maniculatus*) are abundant throughout the parasite range and are traditionally considered the most important intermediate hosts [8, 14]. Furthermore, the competence of southern red-backed vole (*Myodes gapperi*) for *E*. *multilocularis* has been only recently demonstrated [8], and the geographic distribution of the species [15] suggests that this host could be relevant for the transmission of the parasite in Canada. However, local parasite prevalence in small mammals alone cannot illuminate *E*. *multilocularis* transmission unless combined with information on prey selection of the definitive host [16].

Several studies have explored coyote feeding ecology in natural [17], suburban [18–20] and urban areas [21]. Although the relevance of small mammals in coyote diet has been widely documented [22, 23], no research has ever specifically explored the feeding ecology of this opportunistic predator in relation to the transmission of *E*. *multilocularis*. Such a study is particularly relevant in North American urban habitats, where coyotes can be responsible for the maintenance of the urban sylvatic life-cycle of this parasite [19, 21–24]. In particular, data available for Calgary (AB, Canada) would suggest that coyotes are highly more abundant than red foxes inside the city limits, and can thus can act as the main local wild definitive host [13]. To date, studies of coyote diet in urban habitats mainly aimed to investigate the usage of human-associated food sources and inform management of coyote-human conflict [25], whereas aspects of disease ecology are still unexplored.

Herein, we investigated the role of definitive host feeding ecology on *E*. *multilocularis* transmission in urban habitats, using coyotes as the focal species. Specifically, we aimed to i) assess seasonal variations of *E*. *multilocularis* infection in coyotes in relation to their diet; ii) determine the impact of coyote selective consumption of small mammal preys on *E*. *multilocularis* transmission; and iii) evaluate how these processes affect parasite transmission through changes in encounter rate of coyotes with *E*. *multilocularis*.

## Material and Methods

### Ethic statement

Small mammals were captured in the field with lethal traps (Woodstream Museum Special Traps). If necessary, animals were euthanized through cervical dislocation by trained operators. The animal use protocol was approved by the Animal Care Committee of the Faculty of Veterinary Medicine, University of Calgary (protocol number: AC12–0037). Field permit was granted by the City of Calgary and Alberta Government (Provincial Parks).

### Study area and sample collection

The study was conducted in the following five parks and natural areas of the City of Calgary (51°5′N, 114°5′W), Alberta, Canada: Nose Hill Park (NHP), Bowmont (BM), Weaselhead Natural Environment Areas (WSH), Southland Lowlands (SL) and Fish Creek Provincial Park (FCPP) (for details and map, see [23, 26]). Habitat types available in Calgary are primarily represented by grassland, although two major rivers, several creeks and water bodies provide a large amount of riparian habitat, often encompassed in city parks, natural areas and golf courses [13].

Between June 2012 and June 2013, coyote feces were collected on a ten-day sampling schedule following methodologies and protocols previously described [27]. After collection, feces were weighed and stored at -80°C for 72 hrs to inactivate *Echinococcus* spp. eggs [13, 28] and protect operators from potential exposure. Samples were then stored at -20°C until analysis.

In the same study sites, the relative abundance of small mammals was estimated through lethal trapping sessions conducted within a broader investigation on the prevalence of *E*. *multilocularis* in intermediate hosts [29]. Small mammals were captured monthly with Woodstream Museum Special traps deployed along 1–2 ha rectangular grids and with a sampling effort of 200 traps per three nights (for details, see [13]). Relative abundance of the species was assumed to be reflected in their effective capture rate [13], controlling for the number of misfires (number of animal captured divided by the number of active traps, multiplied by 100%), with the exception of the northern pocket gopher (*Thomomys talpoides*). For this species, capture rate was not considered a reliable indicator of relative availability in the study area, given its fossorial activity [30] and the type of traps used, and was consequently removed from the analysis of small mammal abundance. Small mammal species that were captured only occasionally (i.e., <10 occurrences) and were not encountered in coyote diet, were also removed from the analysis.

### Coyote diet analysis

After collection, a subsample (4–6 g) of feces was removed and saved for parasitological and molecular analysis [31]. The remaining portion of the fecal sample was then weighed, hand-washed, and macroscopic fragments isolated using a 500 μm mesh sieve [13] and left to dry at room temperature.

Coyote diet was analyzed following the point-frame method [32]: undigested macro-components were mixed and evenly spread on a glass tray (18x28cm, 15x20cm or 11x16.5cm, depending on the amount of material) with an underlying grid of 50 points equally spaced to allow for systematic sampling [33]. Food items were identified by three trained operators, whose reliability and concordance were previously tested. In particular, operators were evaluated on their ability to correctly identify small mammals through a blind test [34] based on 11 hair samples belonging to the following 8 species: meadow vole, southern red-backed vole (*Myodes gapperi*), house mouse (*Mus musculus*), muskrat (*Ondatra zibethicus*), deer mouse, shrews (*Sorex* spp.), northern pocket gopher and western jumping mouse (*Zapus princeps*). Prey remains were microscopically identified using mammalian hair and skull keys [34]. An archive of locally collected mammalian hair, as well as the zoological collection of the Department of Biological Sciences at the University of Calgary, were used as additional supporting reference for the identification of hairs, bones and skulls. Presence of fruit and vegetation was assessed through the recovery of seeds, berries and leaves [35–37], although plants were not identified taxonomically. Similarly, occurrence of reptile, bird and invertebrate remains was recorded on the basis of diagnostic items (i.e., scales, feathers, exoskeleton), but taxa within each category were not classified. Deer (Odocoileus spp.) and hares (Lepus spp.) were identified at the genus level. Ground squirrels (genus *Urocitellus* and *Ictidomys*) and tree squirrels (genus *Sciurus*) were identified at the species level but grouped together as Sciuridae.

Overall coyote diet was quantified using the frequency of occurrence per scat (i.e., percentage of scats containing a given food item) [38], as well as the frequency of occurrence per item (i.e., percentage of the number of occurrences of a given food item of the total number of occurrences of all food items), which estimates the relative importance of each food item in the animal diet [39]. Items accounting for less than 2% of the scat volume, as estimated by the proportion of grid points occupied by each food item [40], were excluded from the analysis [34].

### Small mammal consumption and encounter rate with *E*. *multilocularis*

Samples containing remains of small mammals were further analyzed to estimate the number of individual preys associated with each coyote scat. First, for each fecal sample, hairs of small mammal species were meticulously separated by hand upon morphological identification, and their total amount weighed at four decimal precision with an Ohaus PA214 scale. Mass of prey hairs in the sample analyzed was extrapolated to the total fecal mass. Undigested prey hair mass was then converted to ingested biomass according to an existing predictive model of prey (house mouse *Mus musculus*) consumed by coyotes [41]. Specifically, we applied the average digestibility recorded along sets of feeding trials differing in the number of preys fed per meal (i.e., 10–50) and meal composition (i.e., with or without ground meat filler) [42]. For the purpose of this study, the indigestibility coefficient reported for house mouse hair (0.0252) was considered representative of vole and mouse-size preys (i.e., genus *Microtus*, *Myodes*, *Peromyscus*, *Zapus*, *Sorex*, and *Thomomys talpoides*) [42]. Finally, the number of preys per coyote scat was estimated dividing the total biomass ingested by the mean mass recorded for each small mammal species in our study area [43].

For each species of small mammal occurring in the diet of coyote, the total number of preys ingested per coyote per season was then estimated as

$$N_{\frac{Prey}{season}}=NPrey_{feces}*\;r_{dej}*d$$

where *NPrey*_*feces*_ is the mean number of ingested small mammal preys estimated per coyote feces; r_*dej*_ is the coyote mean daily defecation rate (0.79 scats/day) observed in feeding tests of coyotes, and reported as independent of consumed biomass [13]; d is the number of days in each three-months season (summer: June-August; fall: September-November; winter: December-February; spring: March-May). For each season, we calculated the proportion (0 to 1) of intermediate hosts within the total number of ingested small mammals using the formula

 [IHs/(IHs + NIHs)]

where IHs and NIHs are, respectively, the number of intermediate and non-intermediate hosts ingested per individual coyote [44]. For the purposes of this study, we considered as intermediate hosts all the species of small mammals reported to be competent for *E*. *multilocularis* in North America and found to be present in the study area, including deer mouse, meadow vole, sourthern-red backed vole and house mouse [13].

Finally, seasonal encounter rate (mean, 95% CI) of coyotes with *E*. *multilocularis* was estimated as

$$N_{Encounters}=\sum_{1}^{k}NIHPrey_{i}*\;p_{IHi}$$

where *N IHPrey*_*i*_ is the total number of ingested preys of the *i* intermediate host species, and *p*_*IHi*_ is the parasite prevalence (mean, 95% CI) in the *i* intermediate host species as observed in the study area during the same time period [15, 45, 46]. In detail, prevalence of *E*. *multilocularis* (95% CI) in intermediate hosts was considered as follows. Meadow vole: summer (0–0.007), fall (0.009–0.017), winter (0.034–0.06), spring (0–0.027); deer mouse: summer (0.009–0.017), fall (0–0.005), winter (0.031–0.062), spring (0–0.026); southern red-backed vole: summer (0–0.028), fall (0.021–0.043), winter (0–0.5) [13]. As prevalence of southern red-backed voles was unknown for spring, the species was not considered when estimating the encounter rate with the parasite in that specific season.

### Statistical analysis

Differences in frequency of occurrence per scat of food items across seasons were tested by Fisher’s exact Chi-square test; multiple comparisons across seasons were taken into account using Bonferroni correction to the type I error threshold [13]. Variations in the capture rate of different small mammal species were tested by Kruskal-Wallis test for *k* independent samples. For each species, differences of relative abundance across seasons were tested by Friedman test for paired samples. For pairwise comparisons, we used Mann-Whitney U-tests for independent samples or the Wilcoxon test for paired ones and applied the Bonferroni correction to the type I error threshold [47]. Following ln-transformation and normalization of data, seasonal variations in the relative abundance of small mammals were tested with a one-way ANOVA and Fisher’s least significant difference (LSD) post-hoc test. Seasonal variations in the proportion of intermediate hosts within the total number of ingested small mammals were tested using the Pearson’s Chi-square test. To test for selective consumption of small mammal species by coyotes, we used Fisher's exact Chi-square [48] calculating, for each species, the proportion of individual preys ingested by coyotes (observed) and comparing it to the proportion of animals captured in the field (expected). Ivlev's electivity index [49] was calculated to measure the degree of coyote selection (positive or negative) for small mammal species. Means and Standard Errors (± SEM) are reported throughout the text, unless otherwise specified. All the analyses were run on SPSS version 20.0 (IBM Corporation, USA).

## Results

### Coyote diet

A total of 251 coyote fecal samples were collected in the five sites (NHP, n = 37; BM, n = 71; WSH, n = 51; SL, n = 38; FCPP, n = 54) and submitted to diet analysis. Among vertebrates, small mammals had the highest frequency of occurrence per scat overall (57.1%) and in each season, followed by hares (20.3%), deer (17.5%), Sciuridae (15.1%) and birds (14.3%). Red fox remains were found in coyote feces in summer and spring, with a total frequency of occurrence of 4.7%. Other species that were detected in less than 5% of the coyote feces included muskrat, porcupine, cat and domestic dog (Table 1). Remains of cattle and skunk were identified in one single occasion (<0.5%; data not shown).

**Table 1:** Frequency of occurrence per scat (Scat) and frequency of occurrence per item (Occur) of food items encountered in coyote feces collected in urban Calgary, AB, between June 2012 and June 2013. N indicate sample size (number of feces analyzed).

	Summer (n = 86)		Autumn (n = 69)		Winter (n = 33)		Spring (n = 63)		Overall (n = 251)	
	Scat	Occur	Scat	Occur	Scat	Occur	Scat	Occur	Scat	Occur
Vertebrates										
Small mammals	65.1	16.3	50.7	17.2	39.4	17.1	62.5	23.0	57.1	18.0
Hare (*Lepus* spp.)	11.6	2.9	17.4	5.9	27.3	11.8	32.8	12.1	20.6	6.5
Deer (Odocoileus spp.)	17.4	4.4	20.3	6.9	36.4	15.8	4.7	1.7	17.5	5.5
Birds	27.9	7.0	11.6	3.9	9.1	3.9	1.6	0.6	14.3	4.5
Sciuridae	30.2	7.6	8.7	2.9	6.1	2.6	6.2	2.3	15.1	4.8
Muskrat	11.6	2.9	-	-	-	-	6.2	2.3	5.6	1.8
Porcupine	4.6	1.2	4.3	1.5	12.1	5.3	1.6	2.6	4.8	1.5
Cat	3.5	0.9	2.9	1.0	-	-	-	-	2.0	0.6
Red fox	4.7	1.2	-	-	-	-	1.6	0.6	2.0	0.6
Dog	1.2	0.3	-	-	3.0	1.3	-	-	0.8	0.3
Invertebrates	5.8	1.5	14.5	4.7	3.0	1.3	3.1	1.1	7.1	2.3
Vegetation	97.7	24.4	82.6	27.9	27.3	11.8	84.4	31.0	81.0	25.6
Fruit	57.0	14.2	37.7	21.1	42.4	18.4	42.2	15.5	52.8	16.7
Anthropogenic	18.6	4.7	7.2	2.5	15.1	6.6	1.6	0.6	10.7	3.4

Small mammals, hares and deer represented the key preys of coyotes, overall accounting for 30% of the total food items (range: 23.6%, summer—44.7%, winter) (Table 1). Frequency of occurrence per scat of small mammals varied across seasons (*X* ^*2*^ = 8.290; df = 3; p = 0.039), with a maximum in summer (65.1%) and a minimum in winter (39.4%), although no differences among seasons were detected once the type I error threshold was corrected to account for multiple comparisons (Bonferroni’s correction, *α*’_(*0*.*05; 6*)_ = 0.0083). An opposite trend was observed for deer, for which the frequency of occurrence per scat also varied across seasons (*X* ^*2*^_*Exact*_ = 16.224; df = 3; p = 0.001), reaching its peak in winter (36.4%) and the minimum in spring (4.7%) (winter *vs*. spring, *X* ^*2*^_*Exact*_ = 16.540; df = 1; all the other comparisons not significant). Occurrence of hare showed a similar seasonal trend (*X* ^*2*^_*Exact*_ = 11.263; df = 3; p = 0.009), with the lowest frequency observed in summer (11.6%) and the highest in spring (32.8%) (summer *vs*. spring, *X* ^*2*^_*Exact*_ = 10.044; df = 1; p = 0.002; all the other comparisons, p >0.0083).

Vegetation (25.6%) and fruit (16.7%) were relevant components of coyote diet in terms of frequency of occurrence per item (Table 1), although vegetation was often present only in small amounts (46.5% of the cases with ≤10% volume).

### Small mammal assemblage and relative abundance

A total of 971 small mammals were captured over 30,200 trap-nights, for an overall capture rate of 5.61% when accounting for trap misfires. Small mammal species included meadow vole (n = 267), southern red-backed vole (n = 71), deer mouse (n = 305), western jumping mouse (n = 32) and shrews (n = 296). Relative abundance (effective capture rate, in %) of small mammals varied across species (Kruskal-Wallis test, *X* ^*2*^ = 70.583; df = 3; p<0.001). Overall, highest relative abundance was recorded for deer mouse (1.53±0.29; median = 1.03; range = 0–10.7), followed by shrews (1.37±0.26; median = 0.72; range = 0–9.7), meadow vole (1.27±0.37; median = 0.28; range = 0–14.4), southern red-backed vole (0.61±0.38; median = 0; range = 0–16.7) and western jumping mouse (0.24±0.12; median = 0; range = 0–5.0). No statistical difference (Mann-Whitney test with Bonferroni corrections for multiple comparisons, p>0.005) was observed between the relative abundance of meadow vole, deer mouse and shrews, as well as between southern red-backed vole and western jumping mouse (all the other comparisons, p<0.001). Overall, small mammal relative abundance varied across seasons (F_3, 43_ = 5.226; p = 0.004), with a maximum during summer (6.84±1.47) and fall (8.39±3.12) and a minimum during winter (2.13±0.33) and spring (1.49±0.29) (Fisher’s LSD post-hoc: summer *vs*. winter, p = 0.026; summer *vs*. spring, p = 0.001; fall *vs*. spring, p = 0.004; all the other comparisons not significant).

### Small mammal consumption and encounter rate with *E*. *multilocularis*

**Fig 1:**
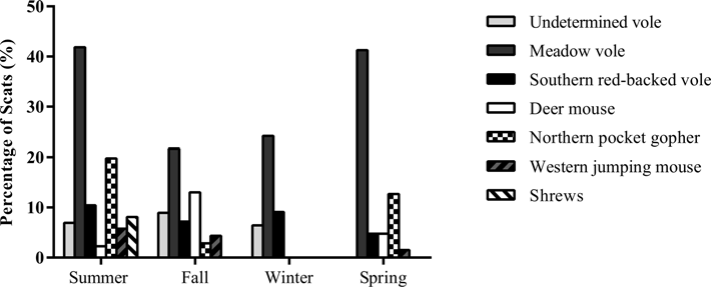
Seasonal variations in the consumption of small mammal species by coyotes, expressed as the percentage of scats containing a given food items (i.e., frequency of occurrence per scat), in five sites in urban Calgary, AB, Canada, between June 2012 and June 2013.

According to the frequency of occurrence per scat, consumption of meadow vole (33.9%) was significantly higher than any other species of small mammals (southern red-backed vole = 8.0%, *X* ^*2*^_*Exact*_ = 50.880, df = 1, p<0.001; deer mouse = 5.6%, *X* ^*2*^_*Exact*_ = 63.428, df = 1, p<0.001; northern pocket gopher = 10.8%, *X* ^*2*^_*Exact*_ = 38.661, df = 1, p<0.001; western jumping mouse = 3.6%, *X* ^*2*^_*Exact*_ = 75.604, df = 1, p<0.001; shrews = 2.8%, *X* ^*2*^_*Exact*_ = 80.969, df = 1, p< 0.001) (Fig. 1). Meadow vole ranked as the most consumed small mammal also according to the mean number of animals ingested per coyote feces (0.90±0.13; median = 0, range = 0–12.44), which was significantly higher than all the other species (southern red-backed vole = 0.39± 0.14; median = 0, range = 0–25.39; *U* = 23597.5, df = 1, p<0.001; deer mouse = 0.17 ± 0.07; median = 0, range = 0–16.54; *U* = 22700.5, df = 1, p<0.001; northern pocket gopher = 0.15 ± 0.04; median = 0, range = 0–3.78; *U* = 23960.5, df = 1, p<0.001; western jumping mouse = 0.08±0.03; median = 0, range = 0–5.84; *U* = 22097.0, df = 1, p<0.001; shrews = 0.02±0.02; median = 0, range = 0–4.60; *U* = 21484.000, df = 1, p<0.001).

Compared to their relative availability, vole species were overall consumed significantly more than expected (meadow vole, *X* ^*2*^_*Exact*_ = 65.288, df = 1, p<0.001; southern red-backed vole, *X* ^*2*^_*Exact*_ = 71.301, df = 1, p<0.001), whereas deer mouse (*X* ^*2*^_*Exact*_ = 82.390, df = 1, p<0.001) and shrews (*X* ^*2*^_*Exact*_ = 161.512, df = 1, p<0.001) were consumed significantly less than expected (Table 2). Positive selection of voles (*M*. *pennsylvanicus* and *M*. *gapperi*) was exhibited in every season with the exception of the fall, during which both species were consumed in proportion of their availability. Deer mouse was preyed less than expected in summer (*X* ^*2*^_*Exact*_ = 64.099, df = 1, p<0.001), winter (*X* ^*2*^_*Exact*_ = 11.745, df = 1, p<0.001) and spring (*X* ^*2*^_*Exact*_ = 47.685, df = 1, p<0.001), but consumed significantly more than expected during fall (*X* ^*2*^_*Exact*_ = 5.527, df = 1, p = 0.023). Shrews were negatively selected during every season (summer, *X* ^*2*^_*Exact*_ = 27.748, df = 1, p<0.001; fall, *X* ^*2*^_*Exact*_ = 36.515, df = 1, p<0.001; winter, *X* ^*2*^_*Exact*_ = 25.686, df = 1, p<0.001; spring, *X* ^*2*^_*Exact*_ = 75.892, df = 1, p<0.001), whereas western jumping mouse was consumed according to its availability with the exception of a significantly higher consumption during fall (*X* ^*2*^_*Exact*_ = 32.897, df = 1, p<0.001) (Table 2).

**Table 2:** Selective foraging of coyotes on small mammal species in urban Calgary, AB, Canada, between June 2012 and June 2013.

	Summer		Fall		Winter		Spring		Overall	
Species	Ivlev	p	Ivlev	p	Ivlev	p	Ivlev	p	Ivlev	p
*Microtus pennsylvanicus*	**0.31**	< 0.001	0.07	0.686	**0.30**	0.043	**0.27**	0.001	**0.28**	< 0.001
*Myodes gapperi*	**0.59**	< 0.001	0.18	0.258	**0.97**	< 0.001	**1.00** ^b^	< 0.001	**0.53**	< 0.001
*Peromyscus maniculatus*	**-0.99**	< 0.001	**0.42**	0.023	**-1.00**	< 0.001	**-0.82**	< 0.001	**-0.54**	< 0.001
*Zapus princeps*	-0.19	0.298	**0.95**	< 0.001	- ^a^	-	1.00^b^	0.559	0.14	0.296
*Sorex* sp.	**-0.73**	< 0.001	**-1.00**	< 0.001	**-1.00**	< 0.001	**-1.00**	< 0.001	**-0.93**	< 0.001

Ivlev's electivity index is calculated for each species using the proportion of individuals ingested (used) and the proportion of animals captured in the field (available); p-values indicate the statistical significance of differences between observed and expected small mammal consumption (Fisher's exact Chi-square test).

^a^ species not captured in the field and not found in feces

^b^ species not captured in the field, but found in feces

Based on the defecation rate applied in this study, we estimated a mean of 116.7±24.2 small mammals consumed per individual coyote per 3-month season, ranging from a maximum of 178.7 during spring to a minimum of 70.6 in winter. Most of the predated small mammals were competent species for *E*. *multilocularis*, resulting in a mean of 101.7±19.8 intermediate hosts ingested per season, and ranging from a maximum of 157.3 in spring to a minimum of 70.6 in winter (Table 3).

**Table 3:** Seasonal and annual estimates of small mammals, overall and infected intermediate hosts (IHs) of *Echinococcus multilocularis* ingested by individual coyotes in Calgary, AB, Canada, between June 2012 and June 2013.

Season	Summer	Fall	Winter	Spring	Total
Small mammals ingested per feces	1.8	1.2	1.0	2.5	1.6
IHs ingested per feces ^a^	1.4	1.1	1.0	2.2	1.4
N small mammals ingested ^b^	130.0	87.4	70.6	178.7	466.7
N IHs ingested ^b^	102.5	76.4	70.6	157.3	406.8
N infected IHs ingested (95% CI) ^c^	0.002 (0.002–1.397)	0.509 (0.496–1.175)	1.052 (0.994–22.443)	0 (0–3.383)	1.563 (1.492–28.398)

^a^ Small mammals reported as susceptible hosts for *E*. *multilocularis* in North America [25].

^b^ Daily defecation rate of coyotes (0.79 scats/day) [15, 45, 46, 50, 51].

^c^ Seasonal prevalence (95% CI) of *E*. *multilocularis* in intermediate hosts in the study area [44].\

**Fig 2:**
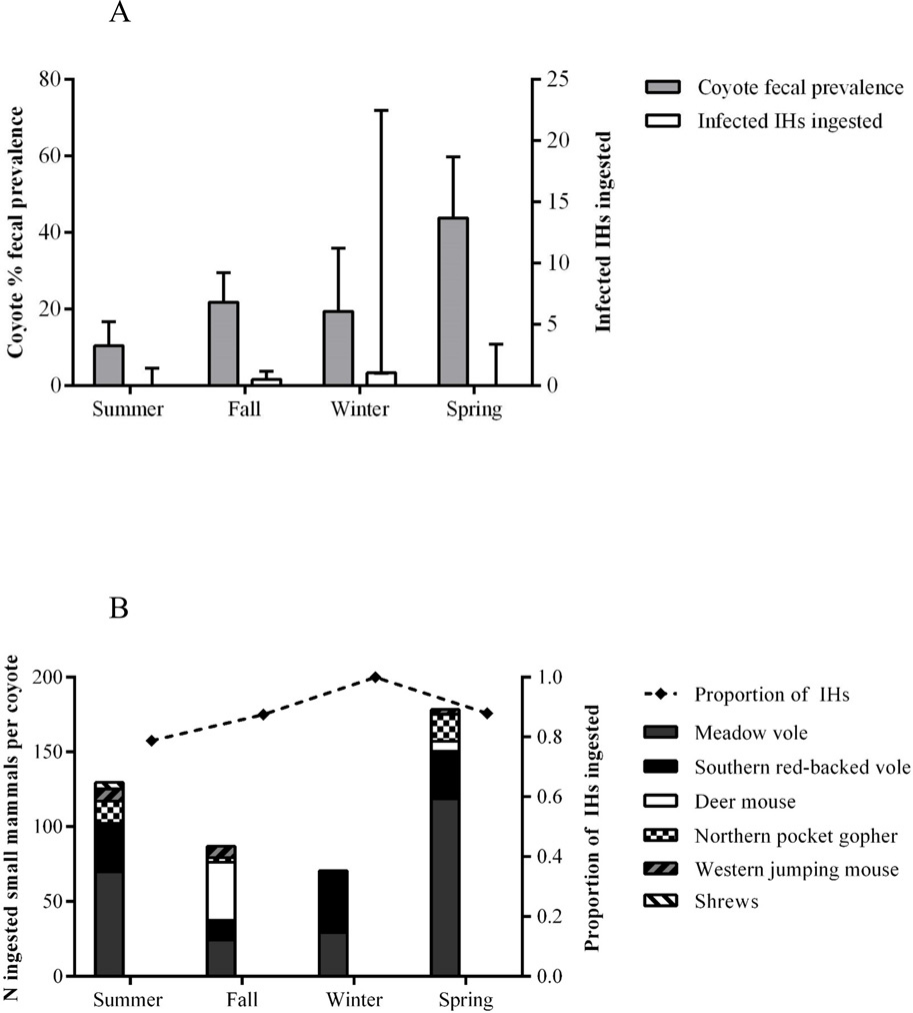
Seasonal variations in coyote fecal prevalence (mean, 95% CI) [13] and the number of infected intermediate hosts (IHs; mean, 95% CI) estimated to be ingested by individual coyote in five sites in urban Calgary, AB, Canada, between June 2012 and June 2013 (A); number of small mammals and proportion of intermediate hosts (IHs/total number of small mammals) estimated to be ingested by individual coyote in five sites in urban Calgary, AB, Canada, between June 2012 and June 2013 (B).

The proportion of intermediate hosts predated varied across seasons (*X* ^*2*^_*Exact*_ = 23.646, df = 3, p<0.001), with that in winter (1.0) being significantly higher than values observed in summer (0.79; *X* ^*2*^ = 17.767, df = 1, p<0.001), fall (0.87; *X* ^*2*^ = 9.649, df = 1, p = 0.003) and spring (0.88; *X* ^*2*^ = 9.148, df = 1, p = 0.001). Meadow voles accounted for the majority of ingested intermediate hosts, with a mean of 60.6±21.9 individuals preyed by coyote per season, and ranging from a minimum of 24.4 in fall to a maximum of 118.8 in spring. Southern red-backed vole was the second most recurrent prey species (mean = 29.6±5.9), followed by deer mouse (11.5±9.3), northern pocket gopher (9.1±4.4), western jumping mouse (4.8±2.0) and shrew (1.1±2.2) (Fig. 2B).

Given the prevalence of *E*. *multilocularis* in intermediate hosts in the study area, the encounter rate with the parasite was highest in winter, with an estimate mean of 1.05 (95% CI: 0.99–22.44) infected intermediate hosts ingested per coyote, and lowest in spring (mean = 0; 95% CI: 0–3.38; Table 3).

## Discussion

Our results suggest that small mammals, fruit and vegetation constituted the bulk of urban coyote diet in the study area, although hare and deer were seasonally very relevant. Within the small mammal assemblage, voles were the selected preys of coyotes and likely played an important role in the transmission of *E*. *multilocularis* in urban Calgary during our study. Despite a relatively lower consumption of small mammals in winter, the encounter rate of coyote with the parasite was at a maximum during this season, when the number of infected intermediate hosts ingested was estimated to be highest.

### Seasonal variations in coyote diet and encounter with *E*. *multilocularis*

In urban Calgary, small mammals were the most frequent prey found in coyote feces in all seasons. For most of the year (summer-winter), their frequency of occurrence in diet reflected their relative availability, as previously documented in prairies [52] and boreal forests [24]. However, this trend was not confirmed for spring, during which we observed a high occurrence of small mammals in coyote diet despite a low capture rate in the field. Due to abundant snow cover, trapping success of small mammals in winter and spring is likely influenced not only by their relative abundance, but also by their activity in the subnivean space [53]. A reduction of the snow cover depth and hardness during spring could expose small mammals to higher risk of predation by coyotes [54, 55] without necessarily increasing their activity above the snowpack and the success of snap-trapping sessions, thus explaining the discrepancy observed.

Interestingly, lower occurrence of small mammals during winter was concurrent with higher consumption of deer. In this season, deer (juveniles in particular) are more exposed to the risk of coyote predation [56], as snow cover and low forage abundance/quality can hinder animal movements and negatively affect their body condition [57]. An increased access of coyotes to alternative (and abundant) food sources could thus concur to explain the decrement in the consumption of small mammals observed in winter. The interdependence between the consumption of small mammals and the availability of deer (i.e., prey switch) has been previously suggested for coyotes [58–60], and could affect the transmission ecology of *E*. *multilocularis* in areas where this canid is the parasite’s main definitive host. Nonetheless, despite a relatively lower number of small mammals predated, encounter rate of coyotes with *E*. *multilocularis* was highest in winter, given the peak in parasite prevalence reported for intermediate hosts in this season [57, 61, 62]. Importantly, composition of local small mammal assemblages can further shape spatial patterns of infection with *E*. *multilocularis* in definitive hosts [13], and our data suggest that such a dilution effect (*sensu* [13]) may be significant in summer but absent in winter, when only competent intermediate host species are consumed (Fig. 2B). These observations are consistent with the higher parasite prevalence documented in coyote feces during spring [63] (Fig. 2A), once the patency period in the canid host (30–90 days post-infection, [13]) is taken into account, further emphasizing the relevance of winter for *E*. *multilocularis* transmission [64].

Our estimates of parasite encounter rate, however, are conservative and need to be interpreted as the minimum number of intermediate hosts ingested per season. Although likely not applicable in urban habitats, higher predation rates on small mammals (and higher defecation rates) were reported for coyotes in Yellowstone National Park [13, 65]. Further research, possibly combining field observation with feeding trials, would allow refining correcting factors for small mammal digestibility and coyote defecation rates. Similarly, larger sample size, multiannual data series and indices of availability for other prey species (i.e., deer, hare) would aid in a better understanding of seasonal variations in coyote diet and parasite encounter rates.

### Coyote diet and *E*. *multilocularis* transmission ecology

Among small mammals, meadow vole and southern red-backed vole were the selected preys of coyotes in the study area. Considering their competence for the parasite [56], they are likely playing a key role in the transmission of *E*. *multilocularis* in Calgary. In particular the meadow vole, given its high abundance, is likely very important for the maintenance of the sylvatic life-cycle of *E*. *multilocularis* in this urban landscape. On the other hand, the southern red-backed vole could be locally highly relevant in relation to suitable wood patches [8, 15], that are in Calgary less abundant and more heterogeneously distributed than grasslands. This species is likely very important in winter, when its contribution to coyote diet and parasite transmission are higher. Although the deer mouse is a locally abundant competent host [66], its under-representation in coyote diet would suggest that it is unlikely to be a key species for the infection of coyotes in urban Calgary.

Microtine multi-annual population fluctuations are considered major drivers of *E*. *multilocularis* transmission intensity [8]. In particular, the relevance of *Microtus* species (i.e. *M*. *arvalis*) for the maintenance of the parasite life-cycle has been recently demonstrated also at large scales [17] where red foxes are the main (and often only) wild definitive host [67], although several other intermediate host species (i.e., *Myodes* sp., *Ellobius* sp., *Ochotona* sp., *Eospalax* sp., *Cricetulus* sp.) can be locally important, depending on ecosystem characteristics [11, 68]. While our data emphasize the importance of considering the whole small mammal assemblage [69, 70], as the relevance of intermediate hosts can vary depending on asynchronous population fluctuations of different species [13], it is necessary to further investigate the interaction between availability of suitable habitats (i.e., ROMPA [17, 69]) and population dynamics of meadow voles in urban habitats, as well as their effect on *E*. *multilocularis* transmission. Integrated with existing knowledge on parasite ecology and epidemiology, such information could help prevent and manage potential disease outbreaks [71].

Finally, our study suggests that coyotes may predate upon red foxes, although occasionally. Such information was not reported in earlier investigations on coyote diet in our study area [72], possibly also due to its rare occurrence. The suppression of red fox populations by coyotes has been documented in North America [38], and it has been hypothesized as the mechanism explaining the low abundance of foxes in urban Calgary [73, 74]. By shaping the composition of the definitive host community and the density of highly susceptible host species such as foxes, the existence of such a direct competition might have consequences on the transmission dynamics of *E*. *multilocularis*. However, our data are not able to tell whether fox is consumed by coyotes as a consequence of predation, direct (interference) competition, or scavenging. Further research is needed to quantify interference competition between coyote and red fox, and assess the relative role of these species (as well as of domestic dogs) in the circulation of *E*. *multilocularis* in Calgary urban and rural habitats.

### Conclusions

In this study we estimated, for the first time, the seasonal encounter rate of a canid host with *E*. *multilocularis* infected intermediate hosts, thus offering a quantitative framework for further epidemiological studies. Our data shows that winter is the most important season for parasite transmission, due to higher encounter rates when coyotes predate upon susceptible intermediate hosts, and the lack of dilution offered by non-competent small mammal species. Furthermore, we provided evidence that within the small mammal assemblage, the meadow vole *Microtus pennsylvanicus* is likely playing a key role in the maintenance of the urban sylvatic life-cycle of *E*. *multilocularis* in Calgary, along with the southern red-backed vole (*Myodes gapperi*) which may be locally very important. Long-term studies of population dynamics of these species, in response to changes in habitat availability and land use, should be integrated in future research on *E*. *multilocularis* transmission in urban habitats to inform disease risk management.
